# Adult Children’s Support and Trajectory of Depressive Symptoms Among Disabled Chinese Older Adults

**DOI:** 10.1093/geroni/igaa057.2705

**Published:** 2020-12-16

**Authors:** Jianyun Wang, Renyao Zhong, Yaolin Pei, Bei Wu

**Affiliations:** 1 East China Normal University, Shanghai, China; 2 New York University, New York, New York, United States

## Abstract

This study aimed to examine the trajectory of depressive symptoms among Chinese older adults with disabilities and the role of adult children’s support in predicting trajectory classes of depressive symptoms. Data were drawn from three waves of the China Health and Retirement Longitudinal Study (2011-2015). The sample included 1420 disabled older adults age 60+ at the baseline and completed all three waves of the data. Growth mixture model shows two-class depressive symptoms trajectories: the higher risk group (25.49%) and the lower risk group (74.51%). Logistic regression results showed that respondents who received a longer term of adult children’s instrumental support were more likely to be classified in a higher risk group after controlling the covariates (OR=1.184, p<0.05), while financial support and the frequency of contacts were not associated with the increased level of depressive symptoms. The policy implications were also discussed in this study.

